# Safety assessment of temporal interference stimulation

**DOI:** 10.3389/fnins.2026.1751719

**Published:** 2026-02-18

**Authors:** Richard Hou, Emma Acerbo, Ryohei Yoshimoto, Nealen Gordon Laxpati, Ken Berglund, Claire-Anne Gutekunst

**Affiliations:** 1 Department of Biology, Emory University, Atlanta, GA, United States; 2 Department of Neurosurgery, Emory University School of Medicine, Atlanta, GA, United States; 3 Wallace H. Coulter Department of Biomedical Engineering, Georgia Institute of Technology, Emory University, Atlanta, GA, United States; 4 Department of Pediatric Neurosurgery, Children’s Healthcare of Atlanta, Atlanta, GA, United States

**Keywords:** astrogliosis, neural safety, neuroinflammation, neuromodulation, thermal injury

## Abstract

Temporal interference (TI) stimulation is a promising non-invasive neuromodulation strategy that uses two high-frequency electric fields to generate a low-frequency amplitude-modulated envelope at their intersection, enabling targeting of deep brain regions. However, *in vivo* safety concerns remain regarding the impact of the low-frequency envelope applied to the brain. Therefore, the objective of this study was to systematically evaluate the acute thermal and cellular safety profile of TI using an invasive *in vivo* mouse model, and to compare its thermal effects with those of direct low-frequency stimulation using an *in vitro* egg-white model. In the egg-white model, no protein coagulation was observed with TI stimulation (10 mA at 1,000 Hz and 1,005 Hz for 20 min), which generated a 5 Hz envelope. In contrast, conventional 5 Hz alternating current stimulation (tACS) at 10 mA induced localized coagulation. In the mouse model, intracranial TI stimulation (2 mA at 1,000 Hz and 1,005 Hz for 20 min) targeting the hippocampus resulted in a mild and stable temperature increase of ∼0.7 °C. Histological analysis revealed a localized increase in astrocyte activation (GFAP) in the stratum lacunosum-moleculare (SLM) compared to other hippocampal subfields. No significant expression difference was observed in the hippocampus for the heat stress marker (HSP70) or the inflammatory marker (iNOS). These findings suggest that TI has a favorable short-term safety profile, with minimal thermal effects and no widespread inflammatory response.

## 1 Introduction

Epilepsy is one of the most prevalent neurological disorders, affecting approximately 50 million people worldwide (Bell and Sander, 2001). While anti-seizure medications are the primary treatment, nearly one-third of patients develop drug-resistant epilepsy, creating a critical need for alternative therapeutic strategies (Kwan et al., 2011). For this population, deep brain stimulation (DBS) has emerged as an effective treatment, but its application is limited by the need for invasive neurosurgery, which carries inherent risks such as hemorrhage and infection (Lozano et al., 2019). Non-invasive brain stimulation, such as transcranial direct current stimulation (tDCS) and transcranial alternating current stimulation (tACS), has sought to provide safer alternatives, but these modalities have historically been limited by a trade-off between focality and depth, often resulting in diffuse stimulation of superficial cortical areas (Simula et al., 2022).

Temporal interference (TI) stimulation was introduced as a non-invasive alternative to overcome this limitation (Grossman et al., 2017). This novel technique leverages a physical principle that two high-frequency (>1 kHz) electric fields (called carrier frequencies) can generate low-frequency modulation transcranially. It has been hypothesized that the individual high-frequency fields oscillate too rapidly for neurons to follow, and instead pass through tissue with lower impedance than low frequency stimulation technology like tACS (<100 Hz). However, at their point of intersection, these high-frequency electrical fields interfere to create a low-frequency amplitude-modulated envelope that oscillates at the difference between the high frequencies. This low-frequency envelope can focally drive neural activity with high spatial precision, offering the hope of DBS-like targeting without invasive surgery (Grossman et al., 2017).

Since the original demonstration of temporal interference stimulation, multiple groups have expanded TI across theoretical, modeling, and *in vivo* systems. Subsequent studies have characterized the biophysical basis and focality of TI using finite-element modeling (Mirzakhalili et al., 2020), optimized electrode configurations and carrier frequencies (Cao and Grover, 2020; Stoupis and Samaras, 2022), and demonstrated modulation of hippocampal and cortical activity in rodent models (Acerbo et al., 2022; Missey et al., 2021). More recent work has extended TI to non-human primates and humans, highlighting both its translational potential and remaining safety considerations (Liu et al., 2024; Violante et al., 2023). Despite these advances, systematic evaluation of tissue-level safety markers under controlled TI conditions remains limited, motivating the present study.

However, safety concerns for TI stimulation stem not only from the high-frequency carriers but also from the low-frequency envelope they create. It is well-documented that low-frequency electrical currents can be harmful. Indeed, they are clinically used in radiofrequency thermocoagulation to intentionally create therapeutic lesions through resistive heating (Bourdillon et al., 2018). While the seminal work by Grossman et al. (2017) included an initial safety screen that found no evidence of apoptosis or DNA damage, it did not specifically investigate the thermal stress profile of the low-frequency envelope. This study directly addresses this critical knowledge gap. We hypothesized that while the low-frequency envelope generated by TI has the potential to cause thermal changes, its indirect generation via interfering high-frequency fields will produce a smaller thermal effect than the direct low-frequency stimulation.

This hypothesis is based on the distinction between a direct 5 Hz tACS current and the TI-generated 5 Hz envelope. tACS provides a true, low-frequency current that must overcome the high, frequency-dependent impedance of biological tissue (*Z*_*HIGH*_), thereby generating significant heat *P* = *I*^2^*Z* (Gabriel et al., 1996). In this equation, *I* is the electrical current passing through the tissue, and *P* is the resulting electrical power released as heat (Joule heating). Because biological tissues act as capacitors, their capacitive impedance (*Z*) is inversely proportional to frequency (ω). Therefore, the low 5 Hz frequency of tACS encounters this *Z*_*HIGH*_. The 5 Hz envelope generated by TI, in contrast, is not a current but rather a temporal pattern of amplitude modulation, which doesn’t follow the *P* = *I*^2^*Z* equation. Consequently, the only true currents propagating through the tissue are the high frequency carriers (>1 kHz). These carriers encounter a much lower tissue impedance (*Z*_*LOW*_), and as a result, should produce minimal heat (Schwan, 1957). This physical distinction allows the 5 Hz neuromodulatory pattern to be effectively decoupled from the large thermal effects of a true 5 Hz current. To test this hypothesis, we performed a multi-modal safety assessment, including *in vitro* thermal-damage models, *in vivo* temperature recordings, and post-stimulation histological analysis.

## 2 Methods

**FIGURE 1:**
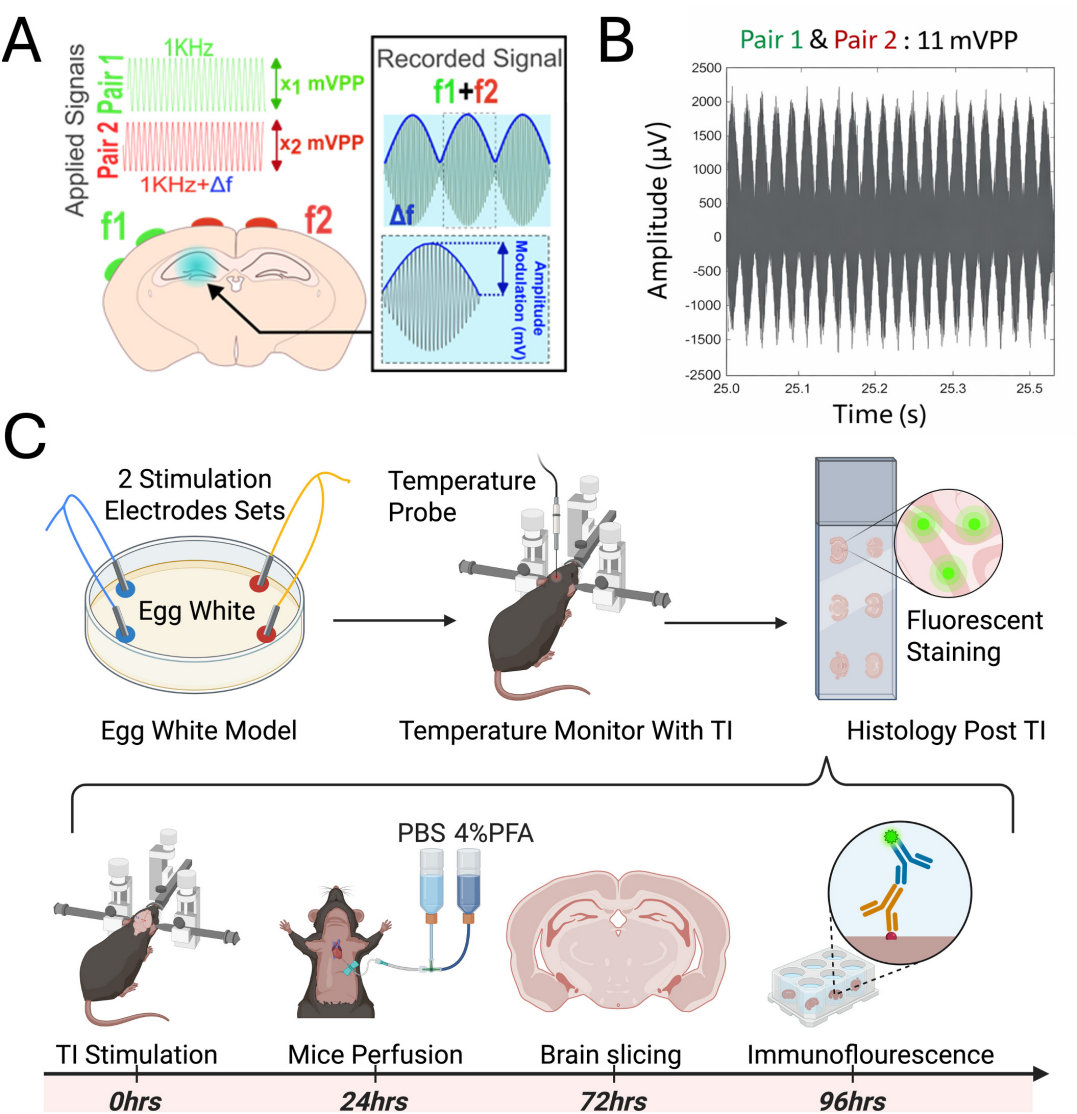
Temporal interference (TI) stimulation paradigm and experimental pipeline. **(A)** Schematic of TI electrode positioning. f_pair1_ = 1 kHz, f_pair2_ = 1.005 kHz, Δf = 50 Hz and all the mVPP values were calculated to reach 2 mV amplitude modulation in the hippocampus. **(B)** Set up and recording during TI stimulation. **(C)** Overall schematic of the experimental pipeline for evaluating the safety of Temporal interference (TI) stimulation.

In this multi-modal approach (Figure 1C), we first used an *in vitro* egg white model to conduct a proof-of-principle test, comparing the thermal coagulation effects of TI stimulation to conventional low-frequency tACS (Figure 1C top). We then moved to an *in vivo* mouse model to measure the intracranial temperature changes in the hippocampus during TI stimulation (Figure 1C top). Finally, a separate cohort of animals underwent the same stimulation paradigm and was sacrificed 24 h later for histological analysis to assess cellular markers for thermal stress (HSP70), inflammation (iNOS), and astrocyte activation (GFAP) (Figure 1C bottom). All stimulation waveforms were continuous and sinusoidal. TI was delivered using 1,000 Hz and 1,005 Hz carriers, selected to match kHz-range carrier frequencies validated in prior rodent TI studies and to remain within hardware limits. tACS was delivered through a single electrode pair because only one low-frequency source is required.

### 2.1 *In vitro* model

An *in vitro* experiment was conducted to assess thermal damage using fresh egg white. Egg white coagulates when its temperature reaches 60 °C and was selected for its protein composition and thermal coagulation properties that are similar to brain tissue (Divkovic et al., 2007; Takegami et al., 2004). The 10 mA amplitude was selected as it is more than what is typically required for transcranial stimulation (Violante et al., 2023), allowing for a robust test of thermal response. Stimulation electrodes were positioned at the edge of a plastic petri dish (5 cm) containing egg white. Stimulation was delivered using independent biphasic stimulus isolators (DS5, Digitimer Ltd., Welwyn Garden City, UK), synchronized through a function generator (EDU33212A, Keysight). The TI group received a 20-min continuous stimulation from two pairs of electrodes (1,000 Hz and 1,005 Hz at 10 mA, *n* = 3), generating a 5 Hz amplitude modulated envelope. The envelope was recorded using an electrode placed in the center of the dish connected to an Intan RHS stim/recording system (Intan Technologies). For comparison, the tACS group was stimulated using one pair of electrodes (5 Hz at 10 mA, *n* = 3) for 20 min. Another tACS group was stimulated at 1 kHz, 10 mA (*n* = 3). For each experiment, the impedance of each pair of electrodes was recorded. Following stimulation, the egg white was inspected visually for protein coagulation. An image of the field was taken at baseline, 10 min and 20 min. For each electrode, the area of coagulation was photographed and quantified using ImageJ^1^ and the coagulated egg white was weighed. The stimulation electrodes were immersed in the egg white sample, and impedance was measured at 1 kHz using a handheld LCR meter (Keysight U1732C, Keysight Technologies, USA) between the current-carrying electrode pair.

### 2.2 Animals

All animal procedures were approved by the Institutional Animal Care and Use Committee (IACUC) at Emory University and were in compliance with all federal regulations. Adult male C57BL/6J mice (*n* = 13) 12–14 weeks of age, weighing 25–30 g were used for experiments. Animals were single-housed following surgical procedures to preserve surgical site integrity. The animal facility was maintained on a 12-h light/dark cycle, and mice had *ad libitum* access to food and water.

### 2.3 Surgery

Mice were anesthetized with isoflurane (3% for induction and 2% for maintenance in oxygen) and secured in a stereotaxic frame. To target the left hippocampus (Missey et al., 2021), four small craniotomies (AP: −1.94, ML: −4.3, −3.3, −0.78, and +0.22 from the bregma; ∼1 mm in diameter) were performed for electrode placement for the target of left hippocampus. The first electrode pair consisted of the two lateral sites (ML: −4.3 and −3.3), and the second pair consisted of the two medial sites (ML: −0.78 and +0.22) (Figure 1A). Electrodes consisted of gold-plated nickel spring-loaded pins (Mill-Max 0950-0-15-20-71-14-11-0) were placed on the dura of the brain. For the temperature monitoring cohort, a fifth craniotomy (AP: −2.7, ML: −2.04, from the bregma; DV: −1.3 from the dura) was made for the insertion of a temperature probe (Cole-Parmer Digi-Sense thermocouple) at a 20° posterior angle (Acerbo et al., 2022, Missey et al., 2021). The thermocouple was calibrated with a two-point water-bath calibration (25 °C and 37 °C). The temperature probe was placed in the predicted TI interference zone. Control animals underwent identical craniotomy without stimulation to control for heat dissipation effects. All coordinates for the electrodes were calculated with the Finite Element Model (Comsol) by Missey et al. (2021), and the electrophysiological confirmation of TI induced neuronal engagement at the hippocampus was verified by Acerbo et al. (2022).

### 2.4 *In vivo* stimulation

While temporal interference (TI) is typically implemented non-invasively using scalp electrodes, early-stage mechanistic and safety studies in rodents frequently employ intracranial configurations to achieve precise field delivery and controlled stimulation geometry. To maintain consistency with these validated paradigms and to enable direct assessment of acute tissue responses, we used an intracranial TI setup in this proof-of-concept study. Stimulation was delivered using two independent biphasic stimulus isolators (DS4, Digitimer Ltd., Welwyn Garden City, UK), synchronized through a function generator (EDU33212A, Keysight). An amplitude of 2 mA was chosen as this is a commonly utilized current for intracranial stimulation in mice given their small brain size (Figure 1B); preliminary testing indicated that higher amplitudes induced twitching of facial muscles, suggesting off-target effects and potential for tissue harm. One cohort of mice underwent intracranial temperature monitoring during a 20-min TI stimulation (1,000 Hz and 1,005 Hz, 2 mA; *n* = 4) and 20-min without stimulation (Sham), temperature was taken through inserted electrode probe with craniotomy. Another cohort for histology was divided into three groups: a TI group receiving 20 min of stimulation (1,000 Hz and 1,005 Hz, 2 mA; *n* = 3); a carrier only group receiving input from two identical 1 kHz waveforms (1,000 Hz and 1,000 Hz, 2 mA for 20 min; *n* = 3) to control for effects of the high-frequency current alone; and a Sham group where electrodes were implanted with no current applied (*n* = 3 for each group).

### 2.5 Histology

Twenty-four hours post-stimulation mice were sacrificed by intraperitoneal injection of pentobarbital/phenytoin mixture and transcardially perfused with ice-cold phosphate-buffered saline (PBS) followed by 4% (w/v) paraformaldehyde (PFA) in PBS. Brains were extracted, post-fixed overnight in 4% PFA in PBS, and then cryoprotected in a 30% (w/v) sucrose in PBS. Coronal brain sections (40 μm thickness) were obtained on a freezing microtome at the level of the hippocampus. Floating sections were immunostained in multi-well plates. Sections were first incubated in a blocking solution [4% (v/v) normal donkey serum in PBS with 0.1% (v/v) Triton X-100] and then with primary antibodies to assess astrocyte activation (rabbit anti-GFAP, 1:1000, Dako, Z0334), thermal stress (rabbit anti-HSP70, 1:1000, Abcam, ab181606), and inflammation (rabbit anti-iNOS, 1:100, Abcam, ab15323). Immunoreactivity was detected with corresponding fluorescently conjugated secondary antibodies (donkey anti-rabbit Alexa Fluor 488, 1:2000, Invitrogen, A32790). Tissue was mounted on glass slides and cover slipped with a mounting medium containing a nuclear counterstain (Vectashield with DAPI, vector lab, H-20000-10).

### 2.6 Microscopy

Images were obtained on a fluorescent microscope (BioTek Cytation 5) with a 4× and 20× objective. To quantify fluorescence intensity, image tiles were collected to cover the entirety of the hippocampus ipsilateral to the stimulation. Quantification was performed in ImageJ software. For each marker, regions of interest (ROIs) were drawn around the stratum lacunosum-moleculare (SLM), dentate gyrus (DG), and CA1 subfields. Each ROI was chosen based on past literature and visual differences. All images were acquired in a single imaging session using identical exposure settings (290 ms), objective, illumination intensity, and acquisition parameters across all samples. ROIs were defined manually within anatomically matched areas (SLM, CA1, DG) using consistent ROI sizes across samples. Background subtraction was performed using subcortical white matter area. No thresholding was applied during quantification, instead, the mean fluorescence intensity was measured for each ROI, averaged and plotted using Prism (GraphPad Software, Boston MA). Each data point represents a per-animal average across 3 sections.

### 2.7 Statistical analysis

All statistical analyses were performed using a statistics software suite in Prism (GraphPad Software, Boston MA). Comparisons of egg white area overtime were performed using a mixed effect model followed by Sidak’s multiple comparisons test. Comparisons of the weight of coagulated egg white were performed using a one-way analysis of variance (ANOVA) followed by Tukey’s *post-hoc* tests. Comparisons of temperature overtime were performed using a mixed effect model followed by Sidak’s multiple comparisons test. Comparisons of fluorescence intensities between the three groups for each hippocampal subfield were performed using a one-way analysis of variance (ANOVA) followed by Tukey’s *post-hoc* tests. A *p*-value of less than 0.05 was considered statistically significant. Data are represented as median with 95% CI.

## 3 Results

### 3.1 TI stimulation did not cause significant thermal effects

**FIGURE 2:**
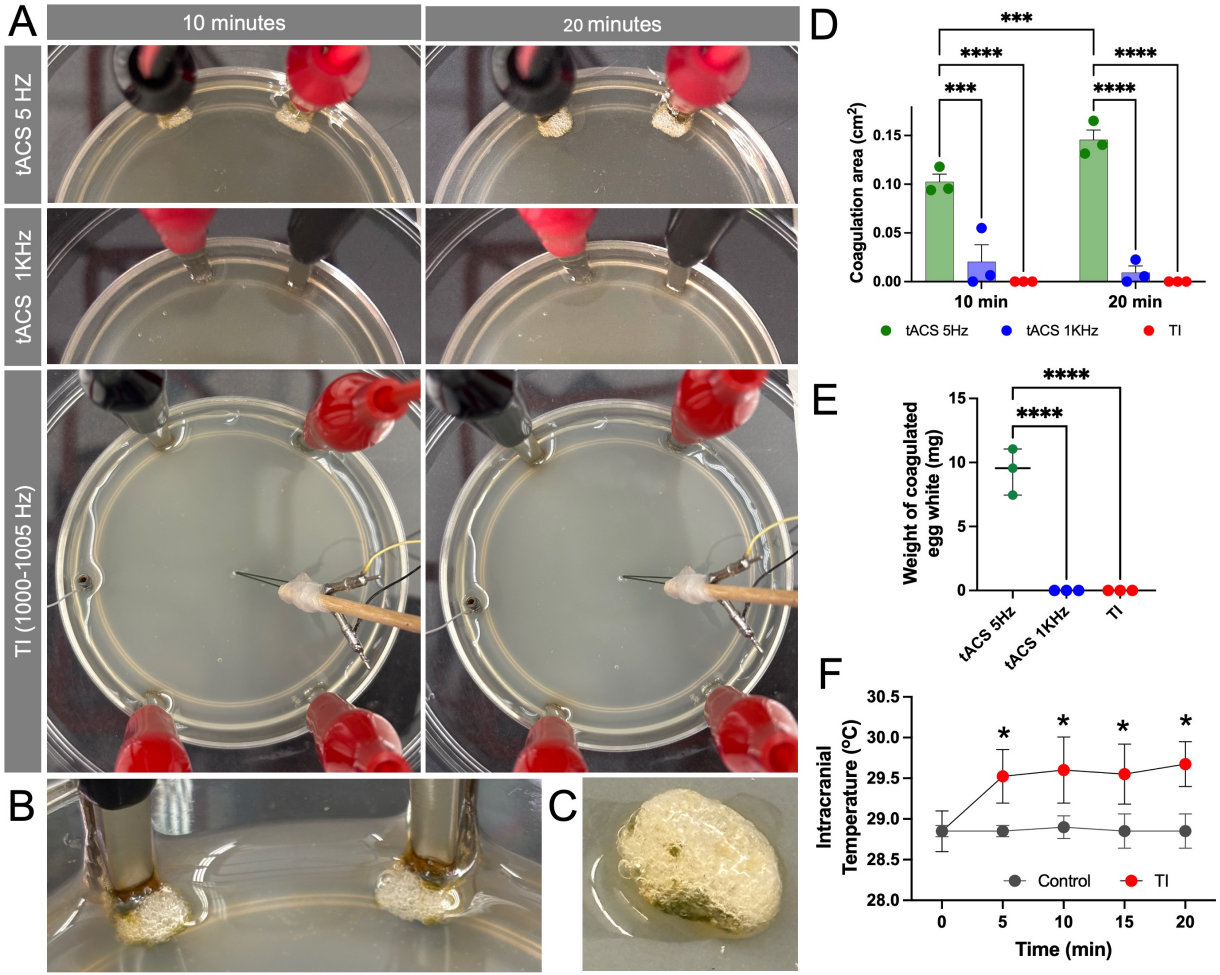
Temporal interference (TI) stimulation did not cause protein coagulation and induced only a mild temperature increase. **(A)** Visualization of the *in vitro* egg white model comparing direct 5 Hz tACS (Top row), 1 kHz tACS (Middle row), and TI (1 + 1.005 kHz) (Bottom row). After 10 and 20 min of stimulation (10 mA), visible protein coagulation is observed only in the direct 5 Hz tACS condition. **(B,C)** A magnified view of the egg white coagulation caused by 5 Hz tACS. **(D)** Quantification of the coagulation area, showing significant coagulation for tACS (5 Hz) compared to minimal or zero coagulation for tACS (1 kHz) and TI at both 10 min and 20 min (***p < 0.001, ****p < 0.0001) (*n* = 3 per group). **(E)** Quantification of the weight of coagulated egg white, showing significant coagulation for tACS (5 Hz) compared to minimal or zero coagulation for tACS (1 kHz) and TI (****p < 0.0001) (*n* = 3 per group). **(F)** Intracranial temperature monitoring in the mouse hippocampus during 20 min of stimulation. TI stimulation induced a stable and mild temperature increase of 0.74 °C ± 0.31 °C compared to the non-stimulated control. Data are represented as median with 95% CI (*p < 0.05) (*n* = 4 per group).

We first assessed the thermal effects of TI and conventional low-frequency stimulation using an *in vitro* model of egg white and an *in vivo* model of mice. tACS at 5 Hz 10 mA resulted in visible protein coagulation in egg white at the stimulation electrode site (Figures 2A–C). In comparison, neither tACS at 1 kHz 10 mA or TI (1–1.005 kHz, 10 mA) producing an equivalent 5 Hz envelope caused any visible coagulation near the stimulation electrode (Figure 2A). In addition, for TI, there was no visible coagulation at the stimulation target site where the envelope was recorded (Figure 2A). Impedance for each set of electrode pairs in egg white ranged between 500 and 720 Ohms. Coagulation area was quantified as area (*p* = 0.00010 at 20 min) and weight (*p* = 0.000047) both demonstrating significant differences between the conditions (Figures 2D, E).

To assess the effect of heat in a more physiologically relevant model, we measured temperature changes in the mouse hippocampus during TI stimulation. In the controlled animal without stimulation, intracranial temperature with craniotomies under anesthesia remained stable at a baseline average of 28.8°C ± 0.1°C over the 20-min recording period. In contrast, TI application resulted in an initial significant increase in temperature (*p* = 0.023), which plateaued after the first 5 min to an average of 29.5°C ± 0.4°C, representing an average increase of 0.74°C ± 0.31°C relative to baseline (Figure 2D). This confirms that while TI is not entirely athermal, the resulting temperature change is minimal and non-escalating.

### 3.2 TI Induced activation of astrocytes localized to the stratum lacunosum-moleculare

**FIGURE 3:**
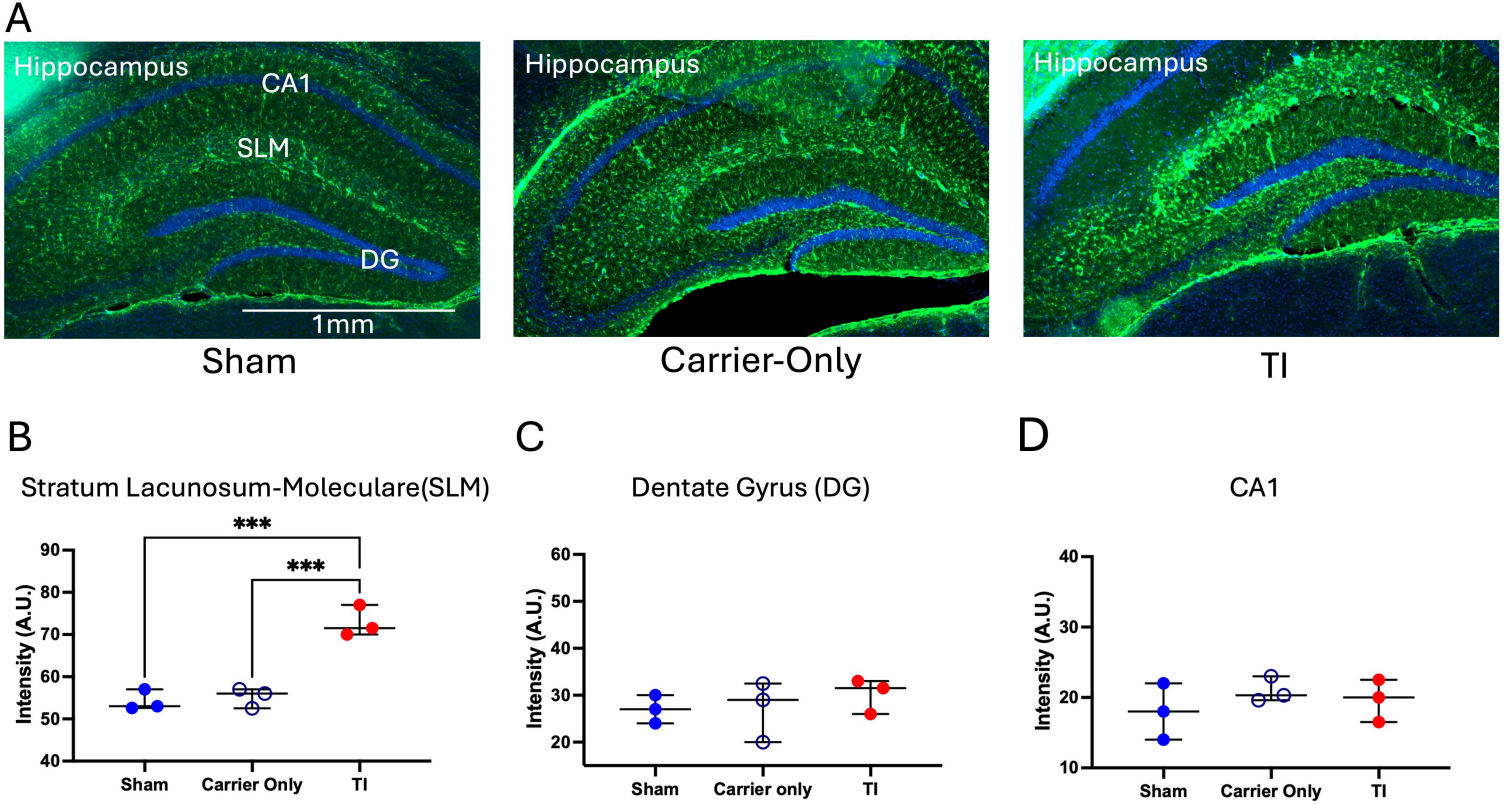
Temporal interference (TI) stimulation induced region-specific astrocyte activation in the hippocampal Stratum Lacunosum-Moleculare (SLM). **(A)** Representative immunofluorescence images of GFAP (green) and DAPI (blue) staining in the hippocampus for Sham (surgical control), Carrier-Only (1 + 1 kHz stimulation), and TI (1 + 1.005 kHz stimulation) groups (*n* = 3 per group). **(B–D)** Quantification of GFAP fluorescence intensity in the stratum lacunosum-moleculare (SLM), dentate gyrus (DG), and CA1. A significant increase in GFAP expression was observed only in the SLM of the TI group (***p < 0.001).

We performed histological analysis on another cohort of mice to assess the cellular response to TI stimulation. In both the Sham and Carrier-only groups, GFAP, a marker for astrocyte activation, showed a widespread low signal in all subregions examined: CA1, DG, and SLM. In contrast, following 20 min of TI stimulation, a highly localized increase in GFAP signal was observed within the SLM subregion (Figure 3A). GFAP levels in the DG (*p* = 0.66,η^2^ = 0.13) and CA1 (*p* = 0.53,η^2^ = 0.19) subregions appeared similar to that in Sham and Carrier-only groups. Quantification of fluorescence intensity confirmed this observation, showing a statistically significant increase of GFAP intensity in the SLM (*p* = 0.00070,η^2^ = 0.93) (Figure 3B), while no significant changes were observed in the adjacent DG or CA1 subfields (Figures 3C, D). This indicates that TI stimulation might cause a spatially restricted astrocytic response.

**FIGURE 4:**
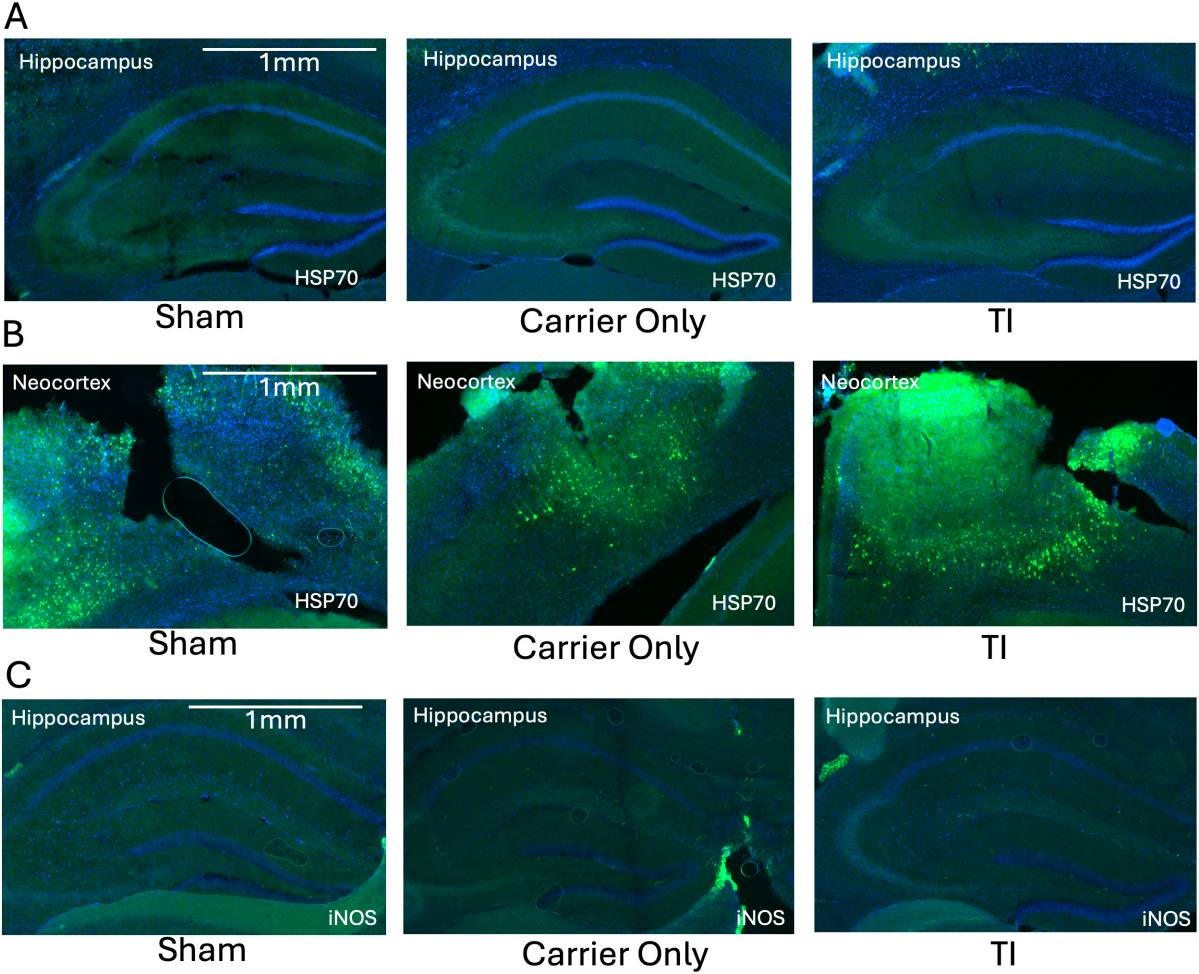
Temporal interference (TI) stimulation did not induce a significant thermal stress or inflammatory response in the hippocampus. **(A)** Representative images showing no detectable HSP70 (green, thermal stress marker) expression in the hippocampus (the stimulation target) across all three experimental groups (*n* = 3 per group). **(B)** In contrast, strong HSP70 expression was observed in the overlying neocortex at the electrode insertion site in all groups, including the surgical Sham, reflecting mechanical injury from electrode insertion. **(C)** Representative images showing minimal and comparable iNOS (green, inflammatory marker) expression in the hippocampus across all groups (*n* = 3 per group). All sections are counterstained with DAPI (blue).

To determine if the mild temperature increase or localized glial activation was associated with broader cellular stress, we examined the expression of HSP70, a heat-shock protein, and iNOS, an inflammatory and vasodilatory marker. HSP70 was not detectable in the hippocampus in any of the groups, indicating that the thermal change after TI was below the threshold required to initiate a cellular heat shock response (Figure 4A). As a positive control, we probed the HSP70 signal in the neocortex immediately below the electrode insertion sites. A strong HSP70 signal was observed, consistent with a mechanical injury caused by the craniotomy and electrode placement (Figure 4B). This signal was not attributable to the high-frequency current itself, as similar HSP70 expression was observed in both the Sham and Carrier-Only groups. Similarly, iNOS expression was minimal in the hippocampus across all groups, with no apparent difference between TI-stimulated animals and the Sham or Carrier-Only groups (Figure 4C). These histological results suggest that TI stimulation did not trigger a significant inflammatory or vasodilatory response in the targeted brain region of the hippocampus.

## 4 Discussion

This study provides a multi-faceted safety evaluation of TI stimulation, specifically addressing the critical question of whether it induces thermal or cellular damage. Our convergent lines of evidence from *in vitro*, *in vivo*, and histological assessments consistently demonstrate that TI, under the parameters tested, exhibits a favorable short-term safety profile and that its neuromodulatory component is effectively decoupled from significant thermal injury.

The *in vitro* egg white experiment served as a proof-of-principle. While direct 5 Hz tACS caused visible protein coagulation, the 5 Hz TI envelope produced no such effect (Figure 2). This result provides initial support for our hypothesis that interfering high-frequency fields produces a smaller thermal effect than direct low-frequency stimulation.

It is important to explain the different current amplitudes used in our *in vitro* and *in vivo* models. The 10 mA current in the egg white model served as a highest amplitude to assess the physical limits of the TI principle, demonstrating that even at this high level, it did not cause thermal coagulation. This 10 mA level was not used in live mice because our preliminary testing confirmed it was biologically unsafe, inducing muscle twitching and the potential for off target effect. We therefore selected 2 mA as the highest amplitude that was well-tolerated by the animals for the *in vivo* safety study. This two-part approach allowed us to first confirm the physics of thermal decoupling *in vitro* (at 10 mA) and then assess the *in vivo* histological safety at a high but biologically tolerated amplitude (2 mA). Due to different impedance and size, transformation of the amplitude utilized on different model should be careful. Human TI studies typically apply 2–4 mA per electrode pair at kHz carrier frequencies and should be considered as a factor when interpreting the result (Missey et al., 2025; Violante et al., 2023).

This was subsequently supported *in vivo*, where we measured only a minor and stable temperature increase of 0.74°C ± 0.31°C (Figure 2D). This thermal load is well within the range of normal physiological fluctuations and is comparable to the<1°C increase reported for other non-invasive techniques like tDCS, which are widely considered safe under low amplitude for clinical use (Khadka et al., 2018). Although an earlier report shows no effect of hippocampal targeted TI stimulation on temperature in the cortex, our study suggests that TI induces minor temperature elevation, within safety ranges, in the target area (Grossman et al., 2017). However, since the animal underwent craniotomy in both control and experimental group, there can be modest difference in thermal and conductive properties that one should be aware.

The histological data further reinforce these findings. First, the complete absence of HSP70 expression in the hippocampus (Figure 4A) confirms the thermal increase was within the safety range, as HSP70 is a highly sensitive marker for temperature increases of 1°C–2°C (Yenari, 2013). Similarly, the lack of iNOS upregulation (Figure 4C) indicates that TI does not trigger a significant neuroinflammatory reaction. Second, our analysis showed no change in GFAP expression within the CA1 and DG subfields (Figures 3C, D). This finding is consistent with the safety screen by Grossman et al. (2017). However, A key contribution of our work is the identification of a novel, highly localized GFAP upregulation specifically within the SLM (Figure 3B), a subregion not a focus by Grossman et al. (2017) GFAP upregulation are often associated with astrogliosis, which is a canonical response to CNS injury (Eng et al., 2000). However, GFAP alone does not definitively establish reactive gliosis, which requires investigation among additional marker and functional assays.

Given the striking absence of corresponding heat shock or inflammatory markers, we argue against the isolated GFAP upregulation represents cellular damage. Instead, we hypothesize that this finding might reflect a circuit-specific physiological response, providing a possible marker for the preferential activation of the entorhinal-hippocampal perforant pathway by the TI stimulation. The SLM is a critical integration hub with extremely high density of synapses, and it is where the distal dendrites of CA1 neurons receive direct input from the entorhinal cortex via the temporoammonic pathway, a circuit fundamental to memory formation (Aksoy-Aksel and Manahan-Vaughan, 2013). This interpretation is strongly supported by recent human studies, which demonstrate that TI targeting the hippocampus also modulates entorhinal cortex activity and enhances memory, confirming functional engagement of this exact circuit (Violante et al., 2023). Therefore, the intense synaptic activity driven by TI in this input-heavy region could place a high metabolic demand on the local astrocytes, causing them to increase their activity. Thus, GFAP expression at the SLM acts more as a physiological, rather than pathological, response (Capogna, 2011; Dvorak-Carbone and Schuman, 1999). Lastly, an alternative, non-mutually exclusive hypothesis is that this finding might reflects functional astrocyte heterogeneity, suggesting SLM astrocytes are intrinsically more sensitive to electrical stimulation than those in other hippocampal layers (Khakh and Sofroniew, 2015). These interpretations remain speculative, and further experiments incorporating additional glial markers, functional assays, and larger cohorts will be required to establish mechanistic conclusions.

Interpretation of GFAP changes following electrical stimulation must also consider the heterogeneity of findings in the existing literature. While the original Grossman et al. (2017) study reported increased c-Fos and GFAP expression following TI, subsequent work has yielded mixed results. For example, Carmona-Barrón et al. (2023) reported inconsistent or reduced immediate-early gene expression following TI, and the preprint Peressotti et al. (2025) suggest that astrocytic responses may depend strongly on stimulation parameters, timing, and tissue context. Similar variability has been reported in studies of tACS and other electrical stimulation modalities (Carmona-Barrón et al., 2023; Monai et al., 2016; Peressotti et al., 2025). In addition, electrode implantation and craniotomy alone can induce local glial and stress responses, including GFAP and heat shock protein expression, which may confound interpretation of stimulation-related effects. While sham controls mitigate this concern, future non-invasive paradigms will be essential for translational relevance.

This study has several limitations. First, due to the small data size, the statistical power is limited, the findings should be interpreted as preliminary and will require confirmation in larger cohorts. Second, our findings are predicated on an acute, single-session stimulation paradigm. As chronic administration is required for most therapeutic applications, future preclinical studies must assess the cumulative effects of long-term TI. Third, our investigation utilized a single parameter set; a systematic exploration of different parameter space (e.g., amplitude, duration, envelope frequency) would fully delineate the therapeutic and safety window. Fourth, the observed increase in GFAP immunoreactivity within the SLM should be interpreted cautiously. GFAP alone does not definitively establish reactive astrogliosis, and one must be aware of potential GFAP and HSP70 changes due to surgical manipulation or electrode implantation. Finally, the isolated GFAP upregulation observed in the SLM, requires more mechanistic investigation. An expanded biomarker panel such as additional astrocytic markers (e.g., S100β), microglial markers (e.g., Iba1), cytokine profiling, and region-specific gene expression analysis could help confirmed the presence of potential localized astrogliosis and pathological stress.

Despite these limitations, our findings provide initial evidence that TI exhibit a favorable acute safety profile both *in vitro* and *in vivo*. TI produced only minimal temperature increases and no detectable induction of HSP70 or iNOS and the upregulation of GFAP was spatially restricted to the SLM.

## Data Availability

The raw data supporting the conclusions of this article will be made available by the authors, without undue reservation.

## Footnotes

1
https://imagej.net/ij/
